# The Emerging Landscape of Respiratory Viral Infections in Immunocompromised Children

**DOI:** 10.3390/cancers18050725

**Published:** 2026-02-24

**Authors:** Paschalis Evangelidis, Elias Iosifidis, Athanasios Tragiannidis, Emmanouel Hatzipantelis, Emmanuel Roilides, Maria Kourti

**Affiliations:** 1 Second Propedeutic Department of Internal Medicine, Hippokration Hospital, Aristotle University of Thessaloniki, 54642 Thessaloniki, Greece; pascevan@auth.gr; 2 Third Department of Pediatrics, School of Medicine, Faculty of Health Sciences, Aristotle University of Thessaloniki, Hippokration General Hospital, 54642 Thessaloniki, Greece; iosifidish@auth.gr (E.I.); roilides@auth.gr (E.R.); 3 Children & Adolescent Hematology-Oncology Unit, Second Department of Pediatrics, School of Medicine, Aristotle University of Thessaloniki, 54124 Thessaloniki, Greece; atragian@auth.gr (A.T.); hatzip@auth.gr (E.H.)

**Keywords:** immunosuppression, pediatric hematology, pediatric oncology, respiratory viral infections, transplantation

## Abstract

Respiratory viral infections (RVIs) have been described traditionally as clinically important infectious complications in pediatric patients with immunosuppression, particularly in those with malignancies (hematological or solid) and recipients of hematopoietic cell or solid organ transplantation. Specifically, advances in the field of cancer therapy and novel immune-based pharmaceuticals have significantly expanded the population of children with prolonged and complex immunosuppression, whereas the widespread use and availability of molecular diagnostics have increased the detection of respiratory viruses. Additionally, these developments have improved the etiologic identification of RVIs, while introducing important challenges in clinical interpretation, mainly in differentiating incidental viral identification from clinically significant diseases. Furthermore, RVIs in immunocompromised children are characterized by heterogeneous and diverse clinical manifestations, with a range from mild upper respiratory tract involvement to severe lower respiratory tract disease, which can lead to substantial morbidity and mortality. Diagnostic strategies in this field are primarily based on nucleic acid amplification tests, requiring careful interpretation because of the possible prolonged viral shedding, co-detection, and overlapping infectious syndromes. Beyond direct clinical consequences, viral detection has an impact on infection control measures, antimicrobial stewardship decisions, and the timing of therapies. In this literature review, we offer an overview of current evidence on the epidemiology, clinical manifestations, diagnostic approaches, and management of RVIs in immunocompromised pediatric populations, underscoring the unmet need for structured, risk-adapted integration of virologic data into pediatric oncology care.

## Simple Summary

Respiratory viral infections are commonly manifested in children with hematological malignancies, solid tumors, and those undergoing hematopoietic or solid organ transplantation. Advances in cancer therapy and the introduction of novel therapeutics have increased the number of children with immune suppression, thereby making respiratory viruses a major clinical challenge. Moreover, modern molecular diagnostic tests can detect respiratory viruses more frequently, even in cases when symptoms are mild or absent, creating several uncertainties concerning the clinical relevance of viral detection and the way that these results should be interpreted and guide patient management. In this literature review, we provide an overview of current evidence on respiratory viral infections in immunocompromised pediatric patients, with a particular focus on epidemiology, clinical manifestations, molecular diagnosis, and how the molecular viral diagnostics can affect clinical decisions such as antibiotic use, infection control, and timing of cancer therapy administration. Therefore, understanding how to interpret viral test results in this context is crucial to improving the outcomes for this vulnerable patient group.

## 1. Introduction

Respiratory viral infections (RVIs) are among the most common causes of infectious syndromes in pediatric patients [1]. A meta-analysis of 186 articles, including more than 152,000 community-acquired pediatric pneumonia cases in the pre-coronavirus disease-19 (COVID-19) era, found that one or more viruses were identified in 55% of them [2]. Respiratory syncytial virus (RSV) (22.7%) and rhinovirus (22.1%) were the most commonly detected viruses. Risk factors for the development of lower respiratory tract infection (LRTI) include mainly the following, among others: prematurity, low birth weight, male sex, and maternal smoking [3]. In immunocompetent children, RVIs are, in the majority of cases, self-limiting, mainly causing mild upper respiratory tract disease [4,5]. On the contrary, in immunocompromised children, RVIs are associated with both severe clinical manifestations, progression to LRTI, poor outcomes, and increased healthcare utilization costs [6]. This is particularly important in children undergoing therapy for hematological malignancies and in recipients of solid organ (SOT) or hematopoietic cell transplantation (HCT) [7,8]. Immunocompromised children represent a heterogeneous population (estimated at 2–3% of children in high-income countries), with diverse underlying conditions and varying degrees of immune dysfunction that confer different levels of vulnerability to respiratory viral pathogens [9].

Over the last decade, the clinical significance of RVIs in pediatric oncology has significantly increased [7]. Advances that have been made in the field of cancer therapy and the introduction and wider availability in clinical practice of novel pharmaceuticals, such as chimeric antigen receptor-T (CAR-T) immunotherapies, have expanded the population of children exposed to prolonged and complex immunosuppression [10]. Specifically, intensive cytotoxic chemotherapy, high-dose corticosteroids, myeloablative conditioning regimens for HCT, and post-transplant immunosuppression are among the major factors contributing to sustained vulnerability to RVIs in the aforementioned population [10]. As a result, RVIs are encountered during several stages of cancer therapy, SOT, and HCT, not limited only to early treatment stages [11].

At the same time, the diagnostic landscape of RVIs has changed over the last few years [12]. Specifically, the widespread implementation and availability of multiplex nucleic acid amplification tests (NAATs) have resulted in frequent detection of respiratory viruses in hospitalized immunocompromised pediatric patients, including children admitted for febrile neutropenia and even those without predominant respiratory clinical symptoms and manifestations [13]. Thus, respiratory viruses are commonly detected in patients admitted to pediatric hematology–oncology wards, raising several questions regarding their clinical importance, infection control measures, and implications for antimicrobial usage [14].

Importantly, the identification of viral nucleic acid by polymerase chain reaction (PCR) in respiratory samples does not necessarily indicate an active infection in children with immunosuppression [15]. In particular, this can be explained by the high sensitivity of the aforementioned molecular tests and prolonged viral shedding, which can lead to identification of the respiratory viruses, even in the absence of clinically significant disease, and should be interpreted in combination with the clinical manifestations, laboratory and radiologic findings, and patient-related risk factors. Moreover, the respiratory virome is dynamic and can interact with bacterial colonization, with an influence on the risk of disease and clinical severity, but as mentioned above, the presence of a virus does not equate to disease [16].

In the post-COVID-19 pandemic era, modified seasonality and overlapping circulation of multiple respiratory viruses have been described. Nevertheless, these epidemiologic shifts might represent transitional phenomena rather than stable long-term patterns, and normalization of traditional seasonal dynamics remains possible [17,18,19]. Although the majority of hospital admissions during these periods involved otherwise healthy children, immunocompromised pediatric patients appear to have been disproportionately affected in terms of disease severity and risk of potential complications [7,20]. Nevertheless, the impact of RVIs extends beyond respiratory morbidity [10]. Indeed, RVIs have been associated with admission to intensive care units (ICUs), need for respiratory support, prolonged hospitalization, and delays or modifications in the administration of cancer-directed therapy [21,22]. Furthermore, in HCT recipients, the presence of active respiratory viral infection may influence the timing of conditioning of the HCT [11]. The risk of RVIs is determined by both the type and duration of immune defects, such as neutropenia, myelosuppression, lymphopenia, hypogammaglobulinemia, and impaired T-cell immunity [23,24]. Children receiving intensive chemotherapy, those early post-HCT, and allogeneic HCT patients with graft-versus-host disease are particularly at high risk [24]. Therefore, the degree of vulnerability to respiratory viral pathogens is not uniform among immunocompromised children and depends on the nature and depth of immune dysfunction. Defects in humoral, cellular, or innate immunity may differentially influence susceptibility, viral clearance, and the risk of progression to severe LRTI.

In this context, given that RVIs represent clinically meaningful events that influence diagnostic strategies, infection control and prevention, and the timing of cancer therapy, we aimed to conduct this current literature review to summarize the current evidence on RVIs in immunocompromised pediatric patients, with a particular focus on pediatric oncology and HCT populations. Emphasis is given on epidemiology, virus-specific clinical relevance, diagnostic approaches applied, and the role of RVIs in febrile neutropenia management.

## 2. Epidemiology and Clinical Syndromes of RVIs in Immunocompromised Children

### 2.1. Epidemiology of RVIs

As mentioned above, RVIs caused by community-acquired respiratory viruses (CARVs) are common in patients with hematological malignancies and in recipients of HCT, cellular therapies, and SOT [7]. Infections are usually caused by rhinovirus, coronavirus, influenza virus, metapneumovirus, parainfluenza virus, RSV, and adenovirus.

**Figure 1:**
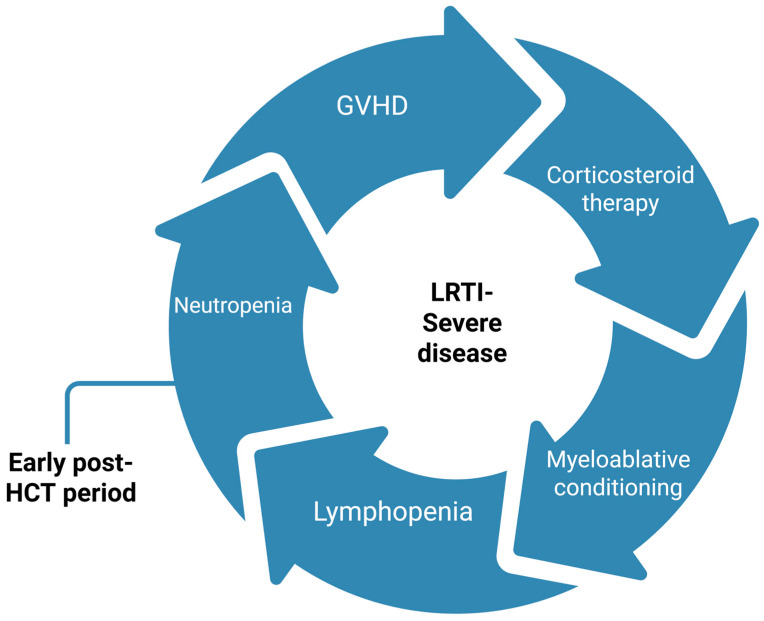
Patient-associated risk factors concerning the development of severe LRTI post-pediatric allogeneic HCT. GVHD: graft-versus-host disease, HCT: hematopoietic cell transplantation, LRTI: lower respiratory tract infection. Created in BioRender. Evangelidis, P. (2026) https://biorender.com/65zaf26, (accessed on 27 January 2026).

In pediatric patients with hematological malignancies, the most common cause of RVIs is rhinovirus [3,25,26]. Similar data are reported in HCT pediatric cohorts. Choi et al. analyzed 402 respiratory specimens obtained from 358 clinical episodes in 116 of 175 pediatric HCT recipients using multiplex reverse transcription PCR [27]. Respiratory viruses were identified in 28.2% of episodes, with the most common being rhinovirus, RSV, and parainfluenza virus. Additionally, a notable percentage of LRTI highlighted the prevalent occurrence and nosocomial acquisition of RVIs in this immunocompromised pediatric population. In a more recently published study, conducted between 2018 and 2021, viral pathogens were detected in 31/64 (48.4%) of upper respiratory tract infection cases (URTI) and 13/23 (56.5%) of LRTI cases, most commonly caused by rhinovirus, followed by severe acute respiratory syndrome coronavirus 2 (SARS-CoV-2), RSV, adenovirus, influenza, and parainfluenza virus [28]. Bacterial and/or fungal co-infections were reported in three patients with LRTIs. The risk factors implicated in the development of LRTI and severe disease in pediatric allogeneic HCT recipients are summarized in Figure 1. Specifically, HCT recipients represent a distinct subgroup characterized by profound and often prolonged T-cell dysfunction, conditioning-related mucosal injury, graft-versus-host disease, and ongoing immunosuppression, factors that may confer a higher risk of progression and viral persistence compared with many children receiving conventional chemotherapy.

In children receiving cellular therapies, such as CAR T-cell products, RVIs represent an increasingly recognized infectious complication [29]. Reported RVI incidence ranges from 8 to 20% within the first 30 days following infusion and increases to more than 50% within the first year after CAR-T cell therapy [29]. Rhinovirus is the most frequently detected viral pathogen, followed by influenza and parainfluenza viruses, underlining mainly the community circulation patterns. Prolonged aplasia of B-cells, hypogammaglobulinemia, and lymphopenia, which may persist for months or years after infusion, are implicated in the pathogenesis of these infections [30]. While pediatric-specific data remain limited, CAR-T cell therapy recipients constitute a recognizable immunocompromised population with prolonged susceptibility to community-acquired respiratory viruses.

In pediatric SOT recipients, available data report that RVIs represent the most common infectious complication after pediatric kidney and liver transplantation, with an estimated frequency of 2.7 episodes per patient-year [31]. As expected, rhinovirus is consistently reported as the most frequently identified pathogen in pediatric SOT recipients, characterized typically by spring and fall peaks, reflecting community circulation patterns. The most detailed pediatric experience derives from lung transplantation, where 13–51% of children develop an RVI within the first months after transplant, most often due to rhinovirus, RSV, adenovirus, or parainfluenza virus [31]. In this setting, younger age and double-lung transplantation have been associated with increased risk, and infections more frequently progress to LRTI compared with immunocompetent children.

Moreover, respiratory virus epidemiology in immunocompromised pediatric patients is influenced by community circulation and seasonality [32]. Post–COVID-19 alterations have been correlated with out-of-season or shifted peaks of RSV and other respiratory viruses in children, which can affect exposure pressure for immunocompromised patients [32]. These modified patterns are of paramount importance to oncology, HCT, and SOT units, with a significant impact on their preparedness and testing triggers tied to “traditional” seasons [7]. It should be highlighted that nosocomial transmission of respiratory viruses is well-documented in pediatric oncology-hematology settings, including even cluster outbreaks [33]. Given that immunocompromised children may shed viruses for prolonged periods, infection control is operationally important in pediatric oncology/HCT wards.

### 2.2. Clinical Syndromes and Disease Progression

In immunocompromised children, respiratory viral infections are mainly presented as URTIs [27,28]. However, progression from URTI to LRTI is a major contributor to severity and poor outcomes in pediatric HCT populations [28]. This progression is strongly affected by patient-associated risk factors, such as lymphopenia, recent allogeneic HCT, early post-transplant period, graft-versus-host disease, and systemic corticosteroid administration, rather than viral species alone [3,34].

Involvement of the lower respiratory tract is the most important predictor of poor outcomes. For instance, in the study of Mowrer and colleagues, pre-HCT rhinovirus infection was not correlated with poor outcomes following transplantation, but children with LRTI had worse survival in comparison to the rest of the study participants [35]. Similarly, this hypothesis has also been validated in a more recent study [36]. In a cohort of pediatric HCT recipients, parainfluenza virus was detected in 46/738 (6.2%) of tested patients, being the most prevalent symptomatic viral infection, with risk factors allo-HCT and total-body irradiation-based conditioning [37]. Among them, 18 (39%) experienced LRTI within the first 100 days post-transplantation, which was associated with steroid use and an absolute neutrophil count <100 cells/μL. As expected, among all the deceased patients (*n* = 6), all had undergone allo-HCT and died due to respiratory failure. In allo-HCT settings, conditioning regimens with total-body irradiation and profound lymphocyte depletion have been correlated with a high risk of respiratory viral LRTI and adverse events, particularly in RSV and parainfluenza infections [38].

Radiographic findings of viral LRTI in immunocompromised patients are, in most cases, non-specific and overlap with other infectious complications, limiting etiologic attribution based on imaging alone. For this reason, the 10th European Conference on Infections in Leukemia (ECIL-10) recommendations emphasize laboratory confirmation and structured diagnostic evaluation when LRTI is suspected in HCT and hematological malignancy patients [7]. However, prolonged NAAT positivity and extended viral shedding can be observed in immunocompromised patients, complicating repeat-test interpretation [39]. Thus, NAAT positivity alone is insufficient to define active disease, and that interpretation should be associated with syndrome (URTI versus LRTI) and the patient’s clinical and laboratory risk factors [7]. Additionally, co-detection and co-infections are clinically relevant in these children [27], whereas in pediatric cancer patients with febrile neutropenia, respiratory virus detection can coexist with bacteremia.

## 3. Respiratory Viruses Beyond SARS-CoV-2

The respiratory viruses affecting immunocompromised children comprise diverse viral families with distinct virological characteristics, including differences in genome structure (RNA vs. DNA viruses), antigenic variability, immune evasion mechanisms, and possibility for LRTI. These biological differences partly explain the heterogeneity in clinical severity, transmissibility, and risk of progression observed across viral species.

### 3.1. Influenza Viruses

Influenza A and B (RNA viruses) cause seasonal epidemics every year, with a variability in epidemiology and severity [40]. Influenza viruses are among the major CARVs relevant not only to patients with malignancies but also to immunocompetent children [7,41]. Specifically, in pediatric HCT cohorts, influenza is repeatedly identified among the detected respiratory viruses and is associated with clinically important morbidity [27,42]. Additionally, influenza viruses have been identified as attributable causes of febrile neutropenia [43]. Early diagnosis is important given that influenza is among the few respiratory viruses for which antiviral therapy is available and most effective when initiated early in the course of infection. However, management should also be supportive, with antivirals recommended for high-risk patients. Neuraminidase inhibitors (oseltamivir, zanamivir) are first-line therapies, which are recommended by ECIL-10, supporting a rapid NAAT-based diagnosis to facilitate timely management in high-risk hematology/HCT patients [7]. Influenza immunization (with an inactivated influenza vaccine) remains central as a primary preventive strategy, while recognizing the potential for reduced immunogenicity in immunosuppressed individuals. Due to the high antigenic variability of influenza viruses because of the continuous antigenic drift and occasional antigenic shift, annual vaccination is essential.

### 3.2. RSV

RSV (RNA virus) is a common pathogen in immunocompetent children, affecting most children before the age of two [44]. RSV is consistently identified as a high-risk respiratory virus in immunocompromised children, primarily due to its strong association with progression from URTI to LRTI [45]. In children with malignancies, an incidence between 1 and 50% has been reported [46]. RSV infection has been described in 0.3–2.2% of pediatric patients with acute myeloid leukemia, in approximately 14% of children with acute lymphoblastic leukemia, and in 8.7% of children with other malignant diagnoses [47]. Particularly, immunocompromised pediatric patients experience an increased prevalence of ICU admission rates and morbidity, with reported mortality ranging from 10 to 19% in pediatric HCT recipients and other high-risk groups with RSV LRTI [48,49]. In patients with RSV, particular emphasis should be placed on syndrome-based stratification (URTI vs. LRTI), as lower respiratory tract involvement is the primary determinant of adverse outcomes and guides escalation of care [7]. Management of RSV infections is generally supportive. Whereas inhaled ribavirin has received approval from the Food and Drug Administration (FDA), it has been associated with several toxicities [23]. Moreover, when administered before mechanical ventilation, ribavirin combined with intravenous immunoglobulin has shown benefit in some high-risk cases; however, evidence remains limited, supporting personalized rather than routine use [50]. Several antiviral drugs are under investigation in clinical trials [51]. One of them, ziresovir, an oral RSV fusion inhibitor, has completed Phase III studies in hospitalized infants (aged <6 months), with favorable efficacy and safety outcomes [52]. Beyond palivizumab (an anti-RSV monoclonal antibody approved for prophylaxis in selected groups), nirsevimab and clesrovimab, extended-half-life monoclonal RSV antibodies, were approved by the FDA in 2023 and 2025, respectively, for all infants entering their first RSV season, including those born during the RSV season. Immunocompromised infants (e.g., those with primary immunodeficiencies) should also receive them during their second year of life. More studies concerning their safety and efficacy in immunocompromised children are crucial and are planned. However, these agents (palivizumab and nirsevimab) are not indicated for the treatment of established RSV infection. The RSVpreF vaccine (Abryvso, Pfizer) is the first FDA-approved vaccine for use during pregnancy to prevent RSV infection in all infants, irrespective of gestational age at birth [53].

### 3.3. Parainfluenza Viruses

Parainfluenza viruses are also causes of clinically important respiratory disease in these populations, due to a high propensity for LRTI development [54]. Up to 40–55% of immunocompromised children with parainfluenza URTI progress to LRTI, with reported mortality up to 37–50% in severe cases [45,55]. Despite this severity, no validated antiviral therapy is available, and, to date, management relies on early detection, supportive care, and infection-control measures [6]. As supported by ECIL-10 recommendations, priority should be given to risk stratification and infection-control interventions over virus-specific treatment strategies for parainfluenza infections [7].

### 3.4. Human Metapneumovirus (hMPV)

hMPV is increasingly described as a relevant pathogen in immunocompromised pediatric patients, with disease manifestations that overlap between those of RSV and influenza [56]. In patients who develop LRTI due to hMPV, the associated mortality can be high (up to 27%) [45]. Beyond an infection in HCT recipients, it can also be a cause of febrile neutropenia [10,13,43]. Despite its clinical impact, no virus-specific therapy is recommended, and its management is supportive.

### 3.5. Rhinovirus/Enterovirus and Endemic Coronaviruses

Rhinovirus remains the most frequently detected respiratory virus in pediatric oncology and HCT cohorts, particularly in episodes of febrile neutropenia evaluated with multiplex PCR [14,57]. Whereas rhinovirus detection is common, clinical outcomes are generally favorable unless LRTI develops, underscoring the importance of syndrome-based interpretation [45]. Nevertheless, in cases of LRTI, a clinical course comparable to RSV and influenza in immunocompromised subjects has been described, reinforcing that rhinovirus is not uniformly benign [45]. Endemic coronaviruses are also commonly detected through multiplex PCR, but usually, their pathogenic significance remains uncertain, and management is supportive [58].

### 3.6. Adenovirus

Human adenoviruses comprise more than 50 different serotypes, with respiratory and gastrointestinal tract involvement being the most common clinical manifestations [59]. Disseminated disease with liver, kidney, and central nervous system involvement can also develop. Immunosuppression represents a significant risk factor for severe adenovirus disease, particularly among patients with profound lymphopenia, T-cell depletion, and those undergoing allo-HCT. Although mortality rates vary according to the type and severity of immune deficiency, severe disseminated adenovirus infection in immunocompromised hosts has been associated with case fatality rates of more than 50% [60]. When an adenovirus is detected in immunocompromised subjects, systemic evaluation and viral load monitoring are suggested [7]. Antiviral therapy with cidofovir has been shown to reduce adenovirus-associated complications in high-risk populations. Brincidofovir has demonstrated antiviral activity and improved safety in pediatric studies, but its clinical use remains restricted, and evidence is largely derived from observational cohorts and compassionate-use settings [61].

Reported mortality associated with respiratory viral infections in hematological patients varies widely by virus and disease phenotype, reaching even to 40% in adenovirus and parainfluenza LRTI, whereas mortality remains substantially lower in isolated URTI [46,62]. An overview of the epidemiology, clinical implications, and outcomes of RVIs in these patients’ groups is given in Table 1.

## 4. Contemporary Diagnosis of Respiratory Viral Infections

### 4.1. Diagnostic Challenges in Immunocompromised Children

The diagnosis of RVIs in immunocompromised children is characterized by several challenges due to the non-typical clinical signs and potential overlap with bacterial and invasive fungal diseases (IFD) [63,64]. Moreover, it is well known that in pediatric oncology and HCT populations, fever may be the only presenting sign, and classic upper respiratory tract signs and symptoms might be absent or attenuated due to immunosuppression [65]. Additionally, respiratory symptoms in children with cancer are often non-specific, and clinical evaluation alone is inadequate to distinguish viral from non-viral etiologies [6]. This is evident in the HCT setting. Specifically, in HCT recipients, LRTI may present with subtle or delayed symptoms, whereas radiographic findings may overlap with those of bacterial pneumonia, IFD, or noninfectious complications such as pulmonary edema, drug toxicity, or lung endothelial injury syndromes [7,66,67].

### 4.2. Molecular Diagnostics as the Cornerstone of Diagnosis

Molecular diagnostics based on NAATs constitute the cornerstone of contemporary RVI diagnosis in immunocompromised children [68]. In this setting, multiplex PCR respiratory viral panels have replaced antigen-based assays to a great extent due to higher sensitivity and rapid turnaround time [6]. In the recently published guidelines by ECIL-10, NAAT-based testing is recommended as the diagnostic standard for CARVs in patients with hematological malignancies and HCT recipients [7]. Multiplex PCR panels allow the detection of multiple respiratory viruses at the same time, including influenza, RSV, parainfluenza virus, rhinovirus/enterovirus, hMPV, adenovirus, and endemic coronaviruses, thereby improving diagnostic performance in immunocompromised populations [69]. Nevertheless, we have to highlight that increased sensitivity does not result in higher clinical relevance per se and that molecular viral testing without structured interpretation may contribute to diagnostic uncertainties [70]. Furthermore, multiplex PCR panels might differ in viral targets, analytical sensitivity, and reporting of thresholds, and clinicians should be familiar with the characteristics of their locally used assays [7]. To achieve this objective, close collaboration among clinicians, clinical and laboratory microbiologists, and other laboratory scientists is crucial.

### 4.3. Specimen Selection and Diagnostic Yield

#### 4.3.1. Upper Respiratory Tract Sampling

Upper respiratory tract specimens (nasopharyngeal swabs or aspirates) constitute the first-line diagnostic approach for suspected RVIs in immunocompromised children [71]. These specimens are minimally invasive and provide high diagnostic performance for most of the aforementioned CARVs [72]. In pediatric oncology and HCT real-world cohorts, nasopharyngeal sampling combined with multiplex PCR has demonstrated high detection rates, even in patients presenting without overt respiratory symptoms [27]. Thus, upper respiratory tract testing is recommended for all immunocompromised patients with respiratory symptoms or unexplained fever during periods of viral circulation [7].

#### 4.3.2. Bronchoalveolar Lavage (BAL) in LRTIs

In cases of suspected LRTI, BAL can offer us a superior diagnostic yield in comparison to upper respiratory specimens [73,74,75]. Specifically, BAL allows direct sampling of the lower respiratory tract and facilitates comprehensive microbiological assessment [76]. ECIL-10 recommends BAL-based NAAT testing for respiratory viruses in immunocompromised patients with radiographic patterns or clinical evidence of LRTI, mainly when upper respiratory samples are negative or inconsistent with clinical findings [7]. However, BAL is invasive and may not be feasible to perform (or may be contraindicated) in all patients, highlighting the potential need for the adoption of a personalized approach.

### 4.4. Interpretation of Results

Interpretation of molecular diagnostics in immunocompromised pediatric patients is an important clinical challenge due to prolonged/persistent viral shedding. NAAT positivity may persist for weeks or even months after symptom resolution, especially for infections caused by rhinovirus, adenovirus, and SARS-CoV-2 [71,77]. We should place emphasis on the fact that the positivity of NAAT alone is not enough to define active infection and should always be interpreted in conjunction with clinical manifestations, symptom onset, and immunosuppression status. In some cases, viral load quantification and cycle threshold values might help us in the interpretation of the results, but they are not standardized and should not be used in isolation for clinical decision-making [6].

Viral co-detection is considered another common issue when multiplex PCR panels are used, especially in immunocompromised children with prolonged viral shedding [27]. Furthermore, as has also been described above, in pediatric patients with malignancies (solid or hematological), respiratory virus detection may coexist with bacteremia or IFDs, underlining that the detection of a respiratory virus does not exclude concurrent serious bacterial infection [13]. Importantly, caution should be used against the over-attribution of clinical disease to respiratory viruses in the absence of supportive clinical or radiologic findings, and to a particular extent in cases of isolated upper respiratory detection [7].

**Figure 2:**
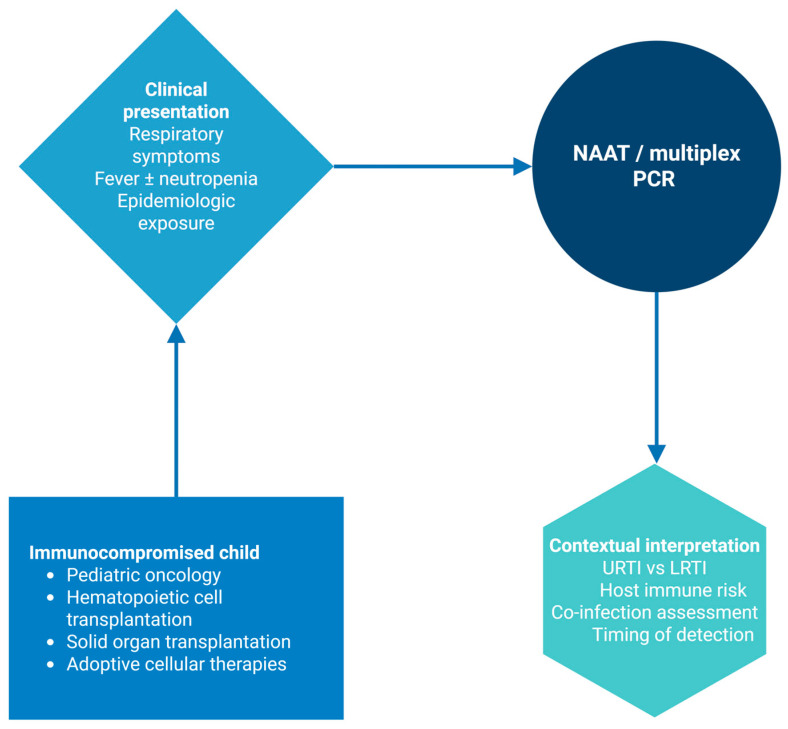
Clinical integration of respiratory viral diagnostics in immunocompromised children. Created in BioRender. Evangelidis, P. (2026) https://biorender.com/cy17vl6, (accessed on 27 January 2026). LRTI: lower respiratory tract; NAAT: nucleic acid amplification tests; PCR: polymerase chain reaction; URTI: upper respiratory tract.

Therefore, viral detection alone does not predict clinical outcomes and should not be used as a “standalone” decision-making tool. In immunocompromised children with suspected severe respiratory infection or LRTI, early escalation is recommended, including lower respiratory tract sampling, comprehensive microbiological evaluation, empiric broad-spectrum antibiotic coverage, and early multidisciplinary team involvement. Diagnostic pathways should combine molecular virologic results with clinical factors (Figure 2).

## 5. RVIs and Febrile Neutropenia: Implications for Antimicrobial Stewardship

### 5.1. RVIs in Febrile Neutropenia: Prevalence and Diagnostics

Respiratory viruses are commonly identified during episodes of febrile neutropenia in children with malignancies, attributed to the wider availability and use of NAAT in everyday clinical practice [5]. In the study of Shinn et al., a large cohort of 404 children with febrile neutropenia (in total 787 episodes) was retrospectively analyzed regarding the epidemiology and outcomes of RVIs [13]. Respiratory viruses were identified using a PCR respiratory viral panel (RVP). In 59% of tested admissions, respiratory viruses were detected, most frequently rhinovirus and enterovirus. RVP positivity was associated with admission to the ICU [odds ratio: 3.19, *p* < 0.002]. Importantly, in the same cohort, concurrent bacteremia was still observed in a subset of admissions despite respiratory virus detection.

Multiplex PCR is characterized by high diagnostic yield during episodes of febrile neutropenia. Real-world viral-distribution data show the predominance of rhinovirus, RSV, and parainfluenza virus among detected pathogens in febrile neutropenia cohorts [6]. A practical implication would be to perform NAAT of the upper respiratory tract to identify a respiratory viral etiology, even in children without profound respiratory symptoms at presentation, which has been reported as a driver for “diagnostic reclassification” over the course of hospitalization [78]. However, the diagnostic capacity of viral molecular testing is affected by various factors, such as sampling timing and the problem of prolonged viral shedding in immunocompromised patients, which can complicate causal attribution of fever [10].

The clinical importance of a positive RVP in febrile neutropenia is heterogeneous, ranging from a coincidental detection to the major contributor of systemic inflammatory symptoms and respiratory complications [6]. Therefore, as has previously been suggested, in pediatric febrile neutropenia, viral detection is clinically useful when integrated into a structured pathway including other clinical parameters: hemodynamic and clinical stability, lack of new clinical signs, and negative bacterial blood cultures [6].

### 5.2. Antimicrobial Stewardship Opportunities: De-Escalation and Safety Boundaries

In pediatric oncology patients with febrile neutropenia, early empiric broad-spectrum antibiotics remain the standard of care because initial clinical presentation may be non-specific and the risk of rapid deterioration from occult bacteremia or sepsis persists, irrespective of respiratory viral detection [79,80]. Importantly, respiratory virus positivity does not exclude concomitant serious bacterial infection: bacteremia and mixed viral–bacterial infections have been reported in febrile neutropenia episodes with a documented respiratory virus [6,13,43]. Within that safety-first framework, respiratory viral diagnostics create a stewardship opportunity mainly through re-evaluation at 48–72 h in carefully selected patients [81]. A pivotal multicenter randomized trial by Santolaya et al. assessed the impact of antimicrobial withdrawal after 48 h in children with malignancies and febrile neutropenia who had demonstrated positivity for a respiratory virus as the only identified pathogen, and a negative bacterial evaluation [82]. A favorable clinical course was found, with similar uneventful resolutions in the withdrawal and continuation arms. Similarly, in a subsequent randomized clinical trial of Torres et al., testing for RVIs led to a statistically significant reduction in antimicrobial therapy duration in children admitted with febrile neutropenia [78].

These trials are clinically important but also highlight a major limitation: stewardship is not “virus-positive,” but rather “virus-positive plus rigorously low-risk (negative blood cultures and clinically stable status) at 48–72 h”. Thus, institutions adopting early discontinuation based on NAAT testing require explicit eligibility criteria, reliable blood culture procedures, and close follow-up to ensure safe patient outcomes.

### 5.3. Implications for Inpatient Oncology Care: Isolation, Infection Control, and Resource Utilization

Beyond antibiotic decisions, respiratory viral detection in pediatric oncology directly has effects on inpatient operational practices, mainly isolation, aiming at the prevention of nosocomial transmission [7]. Isolation practices may be quite demanding, especially in winter seasons when influenza viruses, RSV, and SARS-CoV-2 may co-circulate. In ECIL-10, a common infection-control approach for CARVs in hematologic malignancy/HCT settings is underlined, with a particular emphasis on early identification, appropriate isolation, and heightened measures during outbreaks, principles readily transferable to pediatric hematology-oncology wards [7]. Febrile neutropenia is a high-burden event for families and healthcare systems, and its costs are driven by hospitalization duration, diagnostics, antimicrobial exposure, and isolation-related logistics [6]. As reported in the study of Vargas et al., even modest reductions in the length of inpatient hospitalization days can translate into meaningful system-level savings, which constitute stewardship and relevant molecular viral diagnostics important for healthcare systems [83]. On the other hand, the cost for NAAT performance is also high, as positive results may extend isolation duration and bed-flow constraints, particularly in prolonged positivity of immunocompromised children [84,85].

Collectively, these data indicate that respiratory viral diagnostics in these populations can influence care beyond etiologic identification, interfering with antimicrobial decisions, infection control practices, and inpatient resource utilization. Despite this, their clinical value largely depends on structured interpretation, along with other clinical and laboratory factors.

## 6. Conclusions and Research Agenda for Future Studies

The diagnosis and management of RVIs have become routine and clinically important components of care for immunocompromised pediatric patients, guided by widespread molecular diagnostics and evolving supportive care practices. Nevertheless, the clinical significance of viral detection remains highly context-dependent, with outcomes primarily influenced by patients’ immunosuppression status, site of infection (URTI versus LRTI), and disease severity, rather than by the pathogen itself. This has shifted the diagnostic paradigm from a virus-centered to syndrome- to risk-adapted patient-centered approach, particularly in pediatric oncology and HCT units.

Beyond diagnostics and treatment, vaccination and passive immunization strategies constitute essential components of RVI prevention in immunocompromised pediatric patients. The structured implementation of preventive policies, including vaccination of patients, caregivers, and healthcare workers, is critical to improving outcomes in this vulnerable population.

Contemporary management increasingly requires the integration of virologic molecular data into evidence-based clinical algorithms that balance between safety, antimicrobial stewardship, and infection control.

Key priorities for future research in the field include the following:

- Development of validated pediatric risk stratification models and scores integrating virologic, immunologic, and clinical parameters for the risk stratification of pediatric immunocompromised patients with RVIs. Artificial intelligence and machine learning can be helpful in this approach.
- Clinical Phase 3 studies on novel antiviral agents for the treatment of severe RVIs.
- Randomized trials evaluating virus-informed antimicrobial de-escalation strategies in carefully selected populations.
- A survey investigating practices in this field among several centers worldwide [86]. Additionally, it would be helpful to investigate the availability of NAATs.
- Pediatric-specific efficacy and safety studies of emerging antivirals and preventive agents for high-risk respiratory viruses. Multicenter collaboration is crucial.
- Health systems studies assessing the impact of viral diagnostics on isolation practices and length of stay.
- An individual patient meta-analysis for the investigation of outcomes and risk factors of pediatric HCT recipients who develop RVIs.

## Figures and Tables

**Table 1:** Clinical relevance of major respiratory viruses in immunocompromised children.

Virus	Typical Presentation	Risk of LRTI Progression	Targeted Therapy Availability	Key Clinical Implications
Influenza	URTI ± LRTI	Moderate to high	Yes	Early antiviral therapy, vaccination, and treatment timing are critical
RSV	URTI → LRTI	High	Yes (limited)	High ICU admission risk, prevention strategies
Parainfluenza	URTI → LRTI	High	--	Significant LRTI-associated morbidity
hMPV	URTI ± LRTI	Moderate to high	--	RSV-like disease, supportive care
Adenovirus	Respiratory and systemic	High	Yes	Risk of dissemination, viral load monitoring, and antiviral consideration
Rhinovirus/Enterovirus	Predominantly URTI	Low	--	High prevalence, interpretation with caution, LRTI defines risk

hMPV: human metapneumovirus; ICU: intensive care unit; LRTI: lower respiratory tract infection; RSV: respiratory syncytial virus; URTI: upper respiratory tract infection; → progression to.

## Data Availability

No new data were created or analyzed in this study.

## Abbreviations

The following abbreviations are used in this manuscript:

BAL	Bronchoalveolar lavage
CAR-T	Chimeric antigen receptor-T
CARVs	Community-acquired respiratory viruses
COVID-19	Coronavirus disease 2019
ECIL-10	10th European Conference on Infections in Leukemia
FDA	Food and Drug Administration
HCT	Hematopoietic cell transplantation
hMPV	Human metapneumovirus
ICU	Intensive care unit
IFD	Invasive fungal disease
LRTI	Lower respiratory tract infection
NAAT	Nucleic acid amplification test
PCR	Polymerase chain reaction
RSV	Respiratory syncytial virus
RVI	Respiratory viral infection
RVP	Respiratory viral panel
SARS-CoV-2	Severe acute respiratory syndrome coronavirus 2
SOT	Solid organ transplantation
URTI	Upper respiratory tract infection
